# Simultaneous Determination of Three Compounds in Rat Plasma by UHPLC-QQQ-MS/MS and Its Application to Pharmacokinetics of Banxia Baizhu Tianma Tang

**DOI:** 10.1155/2023/5119997

**Published:** 2023-01-14

**Authors:** Zheming Ying, Guoyuan Sui, Lianqun Jia, Guanlin Yang

**Affiliations:** ^1^The First Clinical College of Liaoning University of Traditional Chinese Medicine, Shenyang, Liaoning, China; ^2^Key Laboratory of Ministry of Education for TCM Viscera-State Theory and Applications, Liaoning University of Traditional Chinese Medicine, Shenyang, Liaoning, China

## Abstract

A rapid and highly selective and sensitive ultra-high-performance liquid chromatography coupled with triple quadrupole mass spectrometry (UHPLC-QQQ-MS/MS) method was applied to simultaneously determine ephedrine, gastrodin, and liquiritin in rat plasma. The three analytes and vitexin-2″-O-rhamnoside (I.S.) were analyzed on a Waters Acquity UPLC C18 column (1.7 *μ*m, 2.1 mm × 100 mm) at 30°C with gradient mobile phase consisting of 0.1% formic acid aqueous solution (A) and acetonitrile (B) after one-step direct protein precipitation with acetonitrile. The detection was performed by multiple reaction monitoring (MRM) mode via electrospray ionization (ESI) source in positive and negative ion modes. The product ions m/z 166.1⟶148.1, 285.1⟶123.1, 417.1⟶255.1, and 579.0⟶433.1 were used for determination of ephedrine, gastrodin, liquiritin, and I.S., respectively. The calibration curves of the three analytes were linear with *r*^2^ greater than 0.994. The intra and interday precision RSD% was less than 11.5 and 13.4. The intra and interday precision RE% was between −10.4% and 9.33%. The average extraction recoveries of the three analytes were no less than 86.88 ± 1.08%. The developed and validated method was for the first time applied to the pharmacokinetics of three compounds in rat plasma after intragastric administration of Banxia Baizhu Tianma Tang.

## 1. Introduction

Banxia Baizhu Tianma Tang originates from the book “Yixue Xinwu” of Qing Dynasty, being the classic prescription of traditional Chinese medicine, which consists of six herbs including *Pinellia ternata* (Thunb.) Breit. (PT), *Atractylodes macrocephala* Koidz. (AM), *Gastrodia elata* Bl. (GE), *Poria cocos* (Schw.) Wolf (PC), *Citrus reticulata* Blanco outer peel (CR), and *Glycyrrhiza uralensis* Fisch. (GU). The formula has the function of drying dampness and resolving phlegm and calming the liver and the wind and is mainly used to treat the wind phlegm dizziness. Modern research reported that the formula has the function of anti-hypertensive and vasodilatory effects [1], and it regulates the blood pressure of spontaneously hypertensive rats and protects the heart [2, 3]. Nowadays, many pharmacological effects of the single herbs in Banxia Baizhu Tianma Tang are found for treating vomiting, coughing and inflammation of PT [4], antitumor, antiviral and anti-inflammatory effects of AM [5] and so on.

Recently, some literature mainly concentrated on in vitro and in vivo the main chemical composition analyses by HPLC [6] and UPLC-Q-TOF-MS [7] methods, and their pharmacological effects of monomer compounds such as ephedrine, gastrodin, liquiritin and etc. in Banxia Baizhu Tianma Tang [8]. To date, some pharmacokinetic studies of the monomer compounds, such as ephedrine [9, 10], gastrodin [11], liquiritin [10, 12] in the single herbs of the formula have been found, but little attention has been devoted to the pharmacokinetic studies of the chemical compositions of Banxia Baizhu Tianma Tang.

The six compounds including ephedrine, gastrodin, atractylenolide, pachymic acid, hesperidin, and liquiritin in Banxia Baizhu Tianma Tang, respectively, represent the main chemical markers for the quality evaluation of PT [13], AM, GE, PC, CR, and GU [14]. Initially, the aim of this study was to investigate the pharmacokinetics of the six chemical compositions in Banxia Baizhu Tianma Tang in rat plasma using the ultra-high-performance liquid chromatography coupled with triple quadrupole mass spectrometry (UHPLC-QQQ-MS/MS) method, but only the pharmacokinetic studies of ephedrine in PT, gastrodin in GE, and liquiritin in GU were investigated at last except atractylenolide, pachymic acid, and hesperidin because only little of them were found in rat plasma. To the best our knowledge, this is first report on the pharmacokinetic studies of ephedrine, gastrodin, and liquiritin in Banxia Baizhu Tianma Tang in rat plasma after intragastric administration in a single dose.

## 2. Materials and Methods

### 2.1. Materials and Chemicals

**Figure 1:**
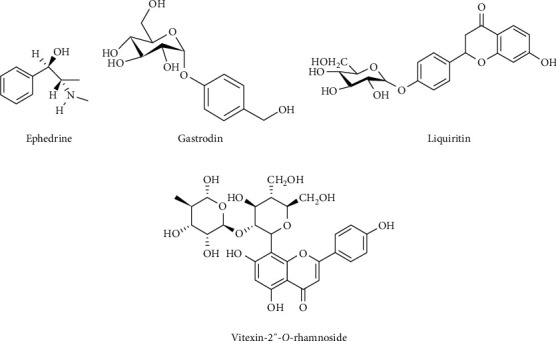
The chemical structures of ephedrine (a), gastrodin (b), liquiritin (c), and I.S. (d).

PT, AM, GE, PC, CR, and GU of Banxia Baizhu Tianma Tang were purchased from the affiliated hospital of Liaoning University of Traditional Chinese Medicine (Shenyang, Liaoning, China) and authenticated by Prof. Rongxiang Wang, a senior expert of herbal authentication at School of Pharmacy, Liaoning University of Traditional Chinese Medicine. The reference standards including ephedrine, gastrodin, liquiritin, and vitexin-2″-O-rhamnoside [15] (internal standard, I.S.) were purchased from the National Institute of Control of Pharmaceutical and Biological Products (Beijing, China). The chemical structures of ephedrine, gastrodin, liquiritin, and I.S. are given in Figure 1. Acetonitrile, methanol (Houston, TX, USA), and formic acid (St. Louis, MO, USA) are all of HPLC grade, and other chemicals and reagents are of analytical grade.

### 2.2. Chromatography and Mass Spectrometry

An UHPLC system (1290 series, Agilent Technologies, Santa Clara, CA, USA) coupled with an Agilent 6460 Triple Quadrupole Mass Spectrometer (QQQ-MS, Agilent Technologies, Santa Clara, CA, USA) equipped with an electrospray ionization (ESI) source operated in positive and negative ionization modes was used to investigate the pharmacokinetic profiles of ephedrine, gastrodin, and liquiritin in Banxia Baizhu Tianma Tang. A Waters Acquity UPLC C18 column (1.7 *μ*m, 2.1 mm × 100 mm, Waters, Milford, MA, USA) at 30°C was used for the chromatographic separations. The mobile phase consists of 0.1% formic acid aqueous solution (A) and acetonitrile (B), and the gradient elution procedure was 5–35% B from 0 to 5 min, 35%–60 B from 5 to 7 min, 60–90% B from 7 to 9 min, and 90% B from 9 to 11 min. An aliquot of 5 *μ*L was injected, and the flow rate was 0.35 mL/min.

The three analytes and I.S. were directly injected into the MS system in order to obtain MS information. The fragmentor voltage (FV) and collision energy (CE) parameters of multiple reaction monitoring (MRM) to detect the three analytes and I.S. were m/z 166.1 ⟶ 148.1 (FV, 90; CE8) for ephedrine, m/z 285.1 ⟶ 123.1 (FV, 100; CE, 4) for gastrodin, m/z 417.1 ⟶ 255.1 (FV, 180; CE, 12) for liquiritin, and m/z 579.0 ⟶ 433.1 (FV200; CE, 16) for I.S. The other parameters were as follows: drying gas (N2) flow rate, 11.0 L/min; drying gas temperature, 300°C; nebulizer pressure, 15 psig; and capillary voltage, 4000 V.

### 2.3. Calibration Standard and Quality Control Samples

Stock solutions of calibration standards including ephedrine, gastrodin, liquiritin, and I.S. were, respectively, prepared in methanol at concentrations of 2.40, 1.25, 5.24, and 1.88 mg/mL. The stock solutions of three analytes and I.S. were serially diluted with methanol to be the working standard solutions of 25.0, 10.0, 5.0, and 1.0 *μ*g/mL in order to prepare calibration curves and quality control (QC) working standard solutions. The final effective concentrations of the calibration standards in rat plasma samples made by spiking 10 *μ*L the corresponding working solutions into 100 *μ*L blank plasma were 2.5, 5, 10, 20, 40, 100, and 250 ng/mL of ephedrine, 50, 100, 200, 400, 1000, and 2500 ng/mL of gastrodin, and 5, 10, 20, 40, 100, 250, and 500 ng/mL of liquiritin. Similarly, the low, middle, and high-concentration QC samples were made by the same way at final concentrations of ephedrine, gastrodin, liquiritin of 5, 40, and 200 ng/mL, 150, 600, and 2000 ng/mL, and 15, 75, and 400 ng/mL, respectively.

### 2.4. Animals and Plasma Sample Preparation

Healthy male SD rats (SCXK2020-0001) (SPF grade, 300 ± 20 g) were purchased from Liaoning Changsheng Biotechnology Co. Ltd. (Shenyang, Liaoning, China). The whole animal experiment process was approved by the Committee of Ethics of Animal Experimentation of Liaoning University of TCM and implemented in strict accordance with the guiding principles of laboratory animal protection. Rats were kept in environmentally controlled breeding room for one week, fed with standard laboratory food as well as water ad libitum, and fasted overnight before the experiments. Aliquots of 100 *μ*L of plasma sample were spiked with 25 *μ*L of I.S. (0.2 *μ*g/mL) and vortex-mixed for 1 min, and then 300 *μ*L of acetonitrile was added to the sample, vortex-mixed for 1 min, and then centrifuged at 3000 rpm for 15 min. The supernatant was transferred into a 1.5 mL EP tube and evaporated to dryness under 40°C of the stream of nitrogen, and 100 *μ*L of methanol was added, vortex-mixed for 1 min, centrifuged at 15,000 rpm for 3 min, and then transferred to an autosampler vial. An aliquot of 5 *μ*L was injected into the UHPLC-QQQ-MS/MS system.

### 2.5. Preparation of Banxia Baizhu Tianma Tang Solution

PT (9 g), AM (18 g), GE (6 g), PC (6 g), CR (6 g), and GU (3 g) in Banxia Baizhu Tianma Tang were, respectively, cut into small pieces, combined and immersed in water for 20–30 min, and then extracted twice with 500 mL water for 1 h and 0.5 h, respectively. The twice extracted solutions were combined, filtered with gauze, and concentrated to about 10 mL under reduced pressure, and then some amount of water was added so that the total volume reached exactly 10 mL that was stored at 4°C before use, and the solution was analyzed by the UHPLC method to obtain the contents of ephedrine, gastrodin, and liquiritin which were 1.699, 0.601, and 0.539 mg/mL, respectively.

## 3. Method Validation

### 3.1. Selectivity and Carry-Over

**Figure 2:**
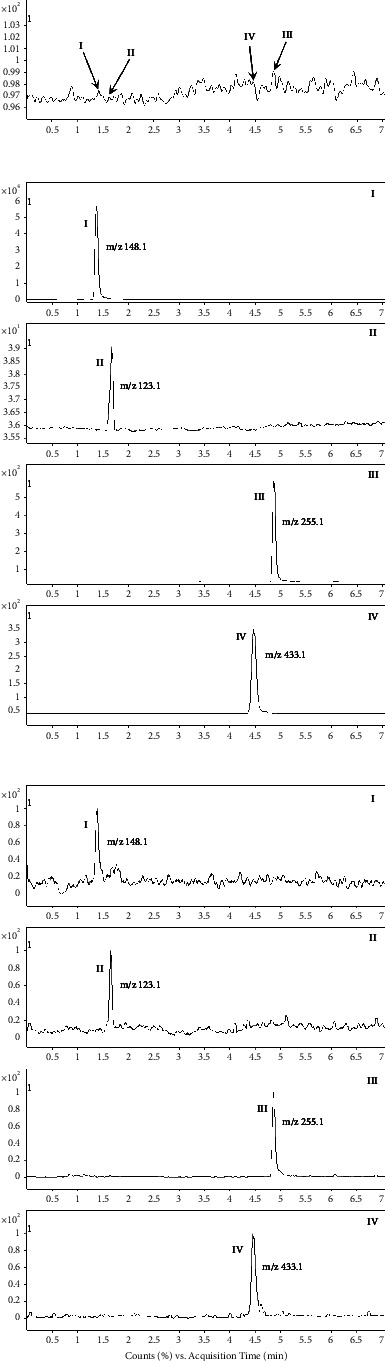
Typical MRM chromatograms of blank plasma (a), a blank sample spiked with the three compounds and I.S. (b), and plasma sample after intragastric administration in a single dose (c) (peak I: ephedrine; peak II: gastrodin; peak III: liquiritin; peak IV: I.S.).

Selectivity was proved by comparing MRM chromatograms of blank plasma obtained from six rats with those of corresponding standard plasma sample spiked with the three analytes and I.S., and plasma sample after administered to the rats via intragastric administration.  Figure 2 shows that the MRM chromatograms of the three analytes and I.S. were free from endogenous matrix interference at their respective retention times.

The cross-talk of ephedrine, gastrodin, and liquiritin as well as I.S. was evaluated by analyzing upper limit of quantification (ULOQ) plasma samples without I.S. and blank plasma samples only with I.S. The responses of the analyte and I.S. of the low limit of quantification (LLOQ) were, respectively, less than 20% and 5%. Carry-over was assessed by injecting blank samples after injecting ULOQ. The results indicated that the carry-over in blank samples following the ULOQ was not greater than 20% of the analyte response at the LLOQ and 5% of the response for the I.S.

### 3.2. Linearity

The calibration curves over the concentration range of 2.5, 5, 10, 20, 40, 100, and 250 ng/mL of ephedrine, 50, 100, 200, 400, 1000, and 2500 ng/mL of gastrodin, and 5, 10, 20, 40, 100, 250, and 500 ng/mL of liquiritin were determined using seven or six standard plasma samples via plotting the peak area ratio of the three analytes to I.S. versus the nominal concentration of the analyte in rat plasma. The regression equations of ephedrine, gastrodin, and liquiritin were obtained using weighted (1/*c*^2^) least squares linear regression: *y* = 0.1642*x* − 0.06984, *y* = 0.0795*x* + 0.06003, and *y* = 0.0051*x* + 0.20200, with a correlation coefficient (*r*^2^) of 0.994, 0.998, and 0.997, respectively, where *y* is the peak area ratio of the analyte to I.S. and *x* is the spiked concentration of the analytes.

### 3.3. Limits of Detection and Quantification

The limits of detection (LOD) were determined in signal to noise ratio (S/N) of 3, which were 0.61, 13.7, and 1.36 ng/mL for ephedrine, gastrodin, liquiritin, respectively. LLOQ was defined as the lowest concentration on calibration curve with acceptable precision and accuracy. LLOQs of ephedrine, gastrodin, and liquiritin were 2.5, 50, and 5 ng/mL (S/N > 10) with RSDs of the intra and interday precisions, respectively, below 11.5% and 13.4% and accuracies (RE%) between −10.4% and 9.11%.

### 3.4. Accuracy and Precision

Precision and accuracy were evaluated by analyzing the QC samples at low, middle, and high concentrations of the three analytes. Precision was expressed as relative standard deviation (RSD%), and accuracy was expressed as (mean found concentration-nominal concentration)/(nominal concentration) × 100%. Intraday precision and accuracy were determined by repeated analysis of a set of standards on one day (*n* = 5), while interday precision and accuracy were determined by repeated analysis on three consecutive days (*n* = 5 series per day). The RSD and RE should be less than 15%, except at the LLOQ where it should not exceed 20% [15]. The data of accuracy and precision for the three analytes from QC samples are listed in Table 1 which indicated that the present method had suitable accuracy and precision [16, 17]; at the same time, the RSDs and REs of the QC samples at LLOQ of the three analytes were less than 20% which met the criteria [17].

### 3.5. Recovery and Matrix Effect

The recoveries were performed by comparing the analytical results of extracted samples at three QC levels with corresponding extracts of blank spiked with the analyte after extraction that represented 100% recovery (*n* = 6). The matrix effects were investigated at three QC levels by comparing the peak areas of post-extraction blank plasma spiked with the three analytes by directly injecting the pure standard solutions at the same concentrations in six replicates (*n* = 6). The matrix effect values should be in the range between 85% and 115%. The recovery and matrix effect of the three analytes are listed in Table 1 meaning that the method had no significant matrix effect and presented high and reproducible recovery.

### 3.6. Stability

The stabilities of QC samples at three concentrations were studied (*n* = 6). Short-term stability and long-term stability studies were determined at room temperature for 4 h and −20°C for 1 month, respectively. Freeze-thaw stabilities were evaluated after three freeze (−20°C)-thaw (room temperature) cycles. Later, the concentrations obtained after samples were processed and analyzed in this experiment were compared with the nominal values of the QC samples. The stability results are listed in Table 1 which demonstrated that the three analytes were stable as no significant degradation of the three analytes in rat plasma occurred under various experimental conditions.

## 4. Results and Discussion

### 4.1. UHPLC-QQQ-MS/MS Optimization

**Figure 3:**
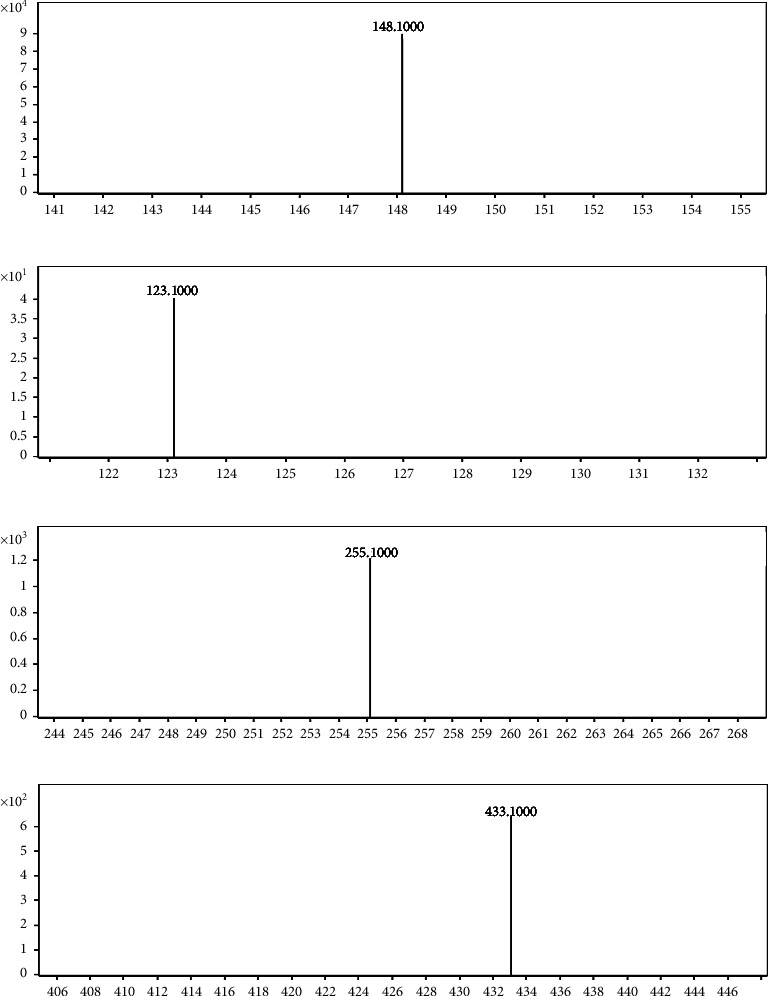
The product ion mass spectra of positive and negative ions of ephedrine (a), gastrodin (b), liquiritin (c), and I.S. (d).

The standard solutions of ephedrine, gastrodin, liquiritin, and I.S. were, respectively, injected into the mass spectrometry system to adjust the instrument setting parameters and maximize the responses. ESI was used in order to obtain good sensitivity and fragmentation. MRM was used for the quantification of the three analytes on account of great advantage in selectivity. Positive ion modes were used to for the quantification of ephedrine and I.S., negative ion modes for that of gastrodin and liquiritin, mainly generated protonated molecules m/z 166.1, 579.0, 285.1 and 417.1 as the precursor ions, respectively. The product ion mass spectra of positive and negative ions of the three analytes and I.S. are shown in Figure 3.

Acetonitrile/methanol-water (containing 0.05%, 0.1%, and 0.2% formic acid) being the mobile phase with gradient elution was used to evaluate UHPLC separation and sensitivity in MS detection. Finally, acetonitrile-water containing 0.1% formic acid, which provides better ionization and higher sensitivity, was selected for the separation of the three analytes and I.S., and the excellent peak shape and mass spectral response were also obtained. Three analytes and I.S. were rapidly eluted with total retention times less than 5.0 min and the whole run time was 7.0 min, in which the retention time of ephedrine, gastrodin, I.S., and liquiritin was 1.43, 1.64, 4.45, and 4.85 min, respectively. Typical MRM chromatograms of a blank sample, a blank sample spiked with three analytes and I.S., and a plasma sample at 30 min after intragastric administration in a single dose are shown in Figure 2.

### 4.2. Pharmacokinetic Application

**Figure 4:**
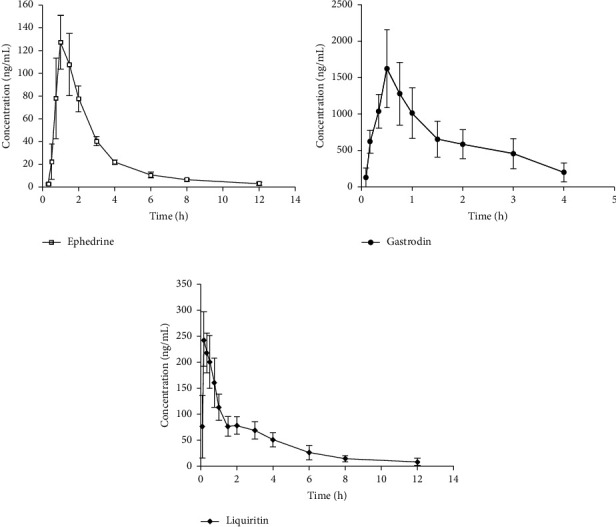
The mean plasma concentration-time curves of ephedrine (a), gastrodin (b), and liquiritin (c) (*n* = 6).

Banxia Baizhu Tianma Tang solution of 16.6 mL/kg approximately containing ephedrine of 28 mg, gastrodin of 10 mg, and liquiritin of 9 mg was administered to the rats via intragastric administration. A blood sample of 0.25 mL was withdrawn from orbital venous at times of 0, 5, 10, 20, 30, 45, 60, 90, 120, 180, 240, 360, 480 and 720 min to heparinized polythene tubes and centrifuged at 3000 rpm for 15 min to obtain plasma samples that were stored at −20°C before analysis. The UHPLC-QQQ-MS/MS method described above was validated successfully and applied to the pharmacokinetic studies of ephedrine, gastrodin, and liquiritin in Banxia Baizhu Tianma Tang. Pharmacokinetic analysis was performed using the DAS 2.0 program (Chinese Pharmacology Society, Beijing, China) to calculate the pharmacokinetic parameters with both compartment and non-compartment approaches. The mean plasma concentration versus time plots of ephedrine, gastrodin, and liquiritin are shown in Figure 4.

### 4.3. Incurred Sample Reanalysis (ISR)

The samples used to prepare the calibration curves and QC during method validation were not the actual study samples because the differences of protein binding, sample in homogeneity or concomitant medications existed and metabolites, which can affect the accuracy and precision of the analyte in such samples during processing and storage. Therefore, incurred samples were reanalyzed of study samples in separate runs at different days to evaluate accuracy of the samples. The samples around the maximum concentration (*C*_max_) and elimination phase for ISR were selected, which could adequate coverage of the entire PK profile. Therefore, 12 samples (2 samples in each rat, *n* = 6) more than 10% of all the 66, 60 and 78 samples of ephedrine, gastrodin and liquiritin were respectively reanalyzed for ISR. Tables 2–4 indicate that the differences between concentrations obtained for the original analysis and the reanalysis were all within 20% of their mean, meaning that 100% ISR met the criteria [16].

In the study, pharmacokinetic analysis was performed using the DAS 2.0 program to calculate the pharmacokinetic parameters with both compartment (a) and non-compartment (b) approaches, a two-compartment open model to gastrodin, and one-compartment open model to ephedrine and liquiritin (weight = 1/*c*^2^) gave the best fit according to the Akaike Information Criterion (AIC) values, and the main pharmacokinetic parameters of the three analytes are listed in Table 5. After intragastric administration, the maximum concentrations (*C*_max_^*b*^) of ephedrine, gastrodin, and liquiritin in plasma were, respectively, 128.2 ± 28.8, 1622.7 ± 657.3, and 244.7 ± 64.2 ng/mL, along with the corresponding values of the area under the concentration-time curves (AUC_0⟶*t*_^a^) of the three analytes were 262.2 ± 42.2, 2483.5 ± 1194.0, and 230.3 ± 135.5 ng h/mL, meaning that ephedrine and liquiritin had a little weak absorption compared with gastrodin. Moreover, both apparent volume of distribution (*V*_*d*_^*a*^) and clearance (CL^*a*^) of ephedrine and liquiritin were larger than those of gastrodin, indicating that ephedrine and liquiritin were mainly distributed in surrounding tissues leading to lower plasma concentrations. In addition, the peak time (*T*_max_^*b*^) of ephedrine, gastrodin, and liquiritin was 0.917, 0.500, and 0.167 h, respectively, suggesting that the three analytes were all rapidly absorbed, in which the liquiritin was the fastest one. The terminal half-life (*t*_1/2_^*b*^) 2.639, 1.278, and 2.176 h, indicated that the eliminations of them were slow. Furthermore, it was noteworthy that ephedrine and liquiritin could be detected up to 720 min, gastrodin up to 240 min after dosed, meaning that they exert a longer lasting effect in vivo in rats, and the advantage of TCM was that the chemical compositions can remain a longer time in body and exert a lasting therapeutic effect and increase the clinical efficacy.

## 5. Conclusions

A developed and validated UHPLC-QQQ-MS/MS method was successfully applied to the pharmacokinetic studies of ephedrine, gastrodin, and liquiritin in rat plasma. Three components in Banxia Baizhu Tianma Tang are simultaneously determined in rat plasma after intragastric administration for the first time. This experiment provides a pharmacokinetic basis for Banxia Baizhu Tianma Tang in clinical aspects and can make Banxia Baizhu Tianma Tang better applied in clinical treatment. Although the pharmacokinetic study of traditional Chinese medicine is very difficult due to its very low content of chemical components in each single herb, ephedrine, gastrodin, and liquiritin which are representative of the herbs of PT, GE, and GU in Banxia Baizhu Tianma Tang can be determined in the study, and further studies will be conducted on other components in this formula in the future.

## Figures and Tables

**Table 1:** Precision, accuracy, recovery, and matrix effect of ephedrine, gastrodin, and liquiritin in rat plasma.

Analytes	Added C (ng/mL)	Extraction recovery (%) (*n* = 6)	Matrix effect (%) (*n* = 6)	Intraday (*n* = 5)			Interday (*n* = 15)			Stability (*n* = 6)		
				Found C (ng/mL)	RSD (%)	RE (%)	Found C (ng/mL)	RSD (%)	RE (%)	Short-term stability (%)	Long-term stability (%)	Freeze-thawstability (%)
Ephedrine	5	86.88 ± 1.08	100.4 ± 3.83	4.57 ± 0.52	11.5	−8.60	4.48 ± 0.60	13.4	−10.4	89.23 ± 2.63	87.43 ± 5.26	87.15 ± 3.81
	40	87.47 ± 2.35	96.40 ± 2.45	41.3 ± 2.8	6.84	3.33	37.6 ± 3.3	8.89	-6.0	91.40 ± 3.05	90.68 ± 2.43	90.04 ± 4.07
	200	92.32 ± 3.69	96.76 ± 3.82	209.8 ± 10.3	4.93	4.90	205.6 ± 8.81	4.28	2.82	91.27 ± 5.22	91.67 ± 1.07	90.66 ± 6.42

Gastrodin	150	89.25 ± 4.07	96.59 ± 1.26	162.7 ± 10.2	6.28	8.4	161.3 ± 18.0	11.1	−7.56	89.29 ± 3.99	90.11 ± 3.78	89.88 ± 2.33
	600	87.98 ± 5.20	96.72 ± 3.57	580.3 ± 20.6	5.15	−3.3	587 ± 35.8	6.09	−2.12	90.69 ± 5.33	90.85 ± 1.04	91.58 ± 1.27
	2000	90.14 ± 3.22	95.15 ± 0.78	1989 ± 66.6	3.35	−0.53	2016 ± 87.2	4.32	−0.80	91.80 ± 4.01	93.11 ± 3.78	93.33 ± 2.63

Liquiritin	15	88.75 ± 6.08	98.17 ± 3.69	16.1 ± 1.7	10.7	7.56	16.4 ± 1.5	9.33	9.11	89.16 ± 1.28	89.23 ± 4.65	88.29 ± 5.96
	75	90.49 ± 3.83	97.71 ± 1.54	70.7 ± 3.3	4.74	−5.78	76.4 ± 1.9	2.46	1.82	91.72 ± 6.47	91.87 ± 1.93	90.59 ± 4.22
	400	90.81 ± 1.73	99.87 ± 7.01	390.8 ± 15.5	3.97	−2.3	389.5 ± 15.0	3.86	−2.63	92.13 ± 1.91	93.33 ± 5.74	92.70 ± 2.91

**Table 2:** Incurred sample reanalysis data of ephedrine.

Subject no.	Sampling point (min)	Original concentration (ng/mL)	Incurred sample concentration (ng/mL)	Mean (ng/mL)	RE (%)
1	30	1.16	1.28	1.22	9.8
1	180	0.401	0.432	0.417	7.4
2	30	1.33	1.49	1.41	11.3
2	180	0.337	0.327	0.332	−3.0
3	30	2.38	2.65	2.52	10.7
3	180	0.730	0.746	0.738	2.2
4	30	1.02	1.12	1.07	9.3
4	180	0.301	0.345	0.323	13.6
5	30	1.58	1.47	1.53	−7.2
5	180	0.426	0.478	0.452	11.5
6	30	1.69	1.81	1.75	6.9
6	180	0.432	0.444	0.438	2.7

**Table 3:** Incurred sample reanalysis data of gastrodin.

Subject no.	Sampling point (min)	Original concentration (ng/mL)	Incurred sample concentration (ng/mL)	Mean (ng/mL)	RE (%)
1	30	1333.3	1351.6	1397.5	1.3
1	120	532.1	538.3	535.2	11.6
2	30	1159.7	1187.5	1173.6	2.4
2	120	371.8	410.9	391.4	10.0
3	30	2375	2422	2398.5	2.0
3	120	852.6	832.5	842.6	−2.4
4	30	1048.1	1008.1	528.1	7.6
4	120	412.8	419.5	416.2	1.6
5	30	1356.3	1387.0	1371.7	2.2
5	120	392.5	412.5	402.5	5.0
6	30	1275	1289	1282	1.1
6	120	367.3	371.4	369.4	1.1

**Table 4:** Incurred sample reanalysis data of liquiritin.

Subject no.	Sampling point (min)	Original concentration (ng/mL)	Incurred sample concentration (ng/mL)	Mean (ng/mL)	RE (%)
1	10	220.1	225.8	223.0	2.6
1	180	64.86	71.2	68.0	9.3
2	10	196.4	216.5	206.5	9.7
2	180	51.7	52.9	52.3	2.3
3	10	317.5	345.8	331.7	8.5
3	180	91.2	82.6	86.9	9.9
4	10	188.5	177.4	183.0	−6.1
4	180	44.39	52.2	48.3	16.2
5	10	154.7	161.9	158.3	4.5
5	180	71.9	84.7	78.3	16.3
6	10	245.4	235.3	235.4	−4.3
6	180	61.2	69.8	65.5	13.1

**Table 5:** Mean pharmacokinetic parameters of ephedrine, gastrodin, and liquiritin in rat plasma after intragastric administration of Banxia Baizhu Tianma Tang solution of 16.6 mL/kg, equivalent to 28 mg/kg of ephedrine, 10 mg/kg of gastrodin, and 9 mg/kg of liquiritin, respectively (mean ± SD, *n* = 6).

Analytes	Parameters						
	*T*_max_ (h)^b^	*C*_max_ (ng/mL)^b^	*t*_1/2_ (h)^b^	*V*_*d*_ (L/kg)^a^	CL (L h/kg)^a^	AUC_0⟶t_ (ng/mL) h^a^	AUC_0⟶∞_ (ng/mL) h^a^
Ephedrine	0.917 ± 0.144	128.2 ± 28.8	2.639 ± 0.574	284.04 ± 52.7	106.6 ± 18.9	262.2 ± 42.2	267.81 ± 43.29
Gastrodin	0.5 ± 0.0	1622.7 ± 657.3	1.278 ± 0.48	4.904 ± 3.171	4.057 ± 2.146	2483.5 ± 1194.0	3012.89 ± 1630.6
Liquiritin	0.167 ± 0.0	244.7 ± 64.2	2.176 ± 0.332	3436.2 ± 1485.4	34.4 ± 14.9	230.3 ± 135.5	314.0 ± 180.8

a and b are the compartmental and non-compartmental approaches, respectively.

## Data Availability

The data used to support the findings of this study are included within the article.
